# Engineering of plants with improved properties as biofuels feedstocks by vessel-specific complementation of xylan biosynthesis mutants

**DOI:** 10.1186/1754-6834-5-84

**Published:** 2012-11-26

**Authors:** Pia Damm Petersen, Jane Lau, Berit Ebert, Fan Yang, Yves Verhertbruggen, Jin Sun Kim, Patanjali Varanasi, Anongpat Suttangkakul, Manfred Auer, Dominique Loqué, Henrik Vibe Scheller

**Affiliations:** 1 Feedstocks Division, Joint Bioenergy Institute, 5885 Hollis St, Emeryville, CA, 94608, USA; 2 Physical Biosciences Division, Lawrence Berkeley National Laboratory, 1 Cyclotron Rd, Berkeley, CA, 94720, USA; 3 Department of Plant Biology and Biotechnology, University of Copenhagen, 40 Thorvaldsensvej, Frederiksberg C, DK-1871, Denmark; 4 Technology Division, Joint Bioenergy Institute, 5885 Hollis St, Emeryville, CA, 94608, USA; 5 Sandia National Laboratories, 7011 East Avenue, Livermore, CA, 94550, USA; 6 Department of Genetics, Faculty of Science, Kasetsart University, Bangkok, 10900, Thailand; 7 Life Sciences Division, Lawrence Berkeley National Laboratory, 1 Cyclotron Rd, Berkeley, CA, 94720, USA; 8 Department of Plant & Microbial Biology, University of California, Berkeley, CA, 94720, USA

**Keywords:** Xylan, Irregular xylem mutant, Secondary cell wall, VND6, VND7, Transcription factors, Biofuels, Pentoses, Saccharification, Lignin

## Abstract

**Background:**

Cost-efficient generation of second-generation biofuels requires plant biomass that can easily be degraded into sugars and further fermented into fuels. However, lignocellulosic biomass is inherently recalcitrant toward deconstruction technologies due to the abundant lignin and cross-linked hemicelluloses. Furthermore, lignocellulosic biomass has a high content of pentoses, which are more difficult to ferment into fuels than hexoses. Engineered plants with decreased amounts of xylan in their secondary walls have the potential to render plant biomass a more desirable feedstock for biofuel production.

**Results:**

Xylan is the major non-cellulosic polysaccharide in secondary cell walls, and the xylan deficient *irregular xylem* (*irx*) mutants *irx7*, *irx8* and *irx9* exhibit severe dwarf growth phenotypes. The main reason for the growth phenotype appears to be xylem vessel collapse and the resulting impaired transport of water and nutrients. We developed a xylan-engineering approach to reintroduce xylan biosynthesis specifically into the xylem vessels in the Arabidopsis *irx7*, *irx8* and *irx9* mutant backgrounds by driving the expression of the respective glycosyltransferases with the vessel-specific promoters of the *VND6* and *VND7* transcription factor genes. The growth phenotype, stem breaking strength, and *irx* morphology was recovered to varying degrees. Some of the plants even exhibited increased stem strength compared to the wild type. We obtained Arabidopsis plants with up to 23% reduction in xylose levels and 18% reduction in lignin content compared to wild-type plants, while exhibiting wild-type growth patterns and morphology, as well as normal xylem vessels. These plants showed a 42% increase in saccharification yield after hot water pretreatment. The *VND7* promoter yielded a more complete complementation of the *irx* phenotype than the *VND6* promoter.

**Conclusions:**

Spatial and temporal deposition of xylan in the secondary cell wall of Arabidopsis can be manipulated by using the promoter regions of vessel-specific genes to express xylan biosynthetic genes. The expression of xylan specifically in the xylem vessels is sufficient to complement the *irx* phenotype of xylan deficient mutants, while maintaining low overall amounts of xylan and lignin in the cell wall. This engineering approach has the potential to yield bioenergy crop plants that are more easily deconstructed and fermented into biofuels.

## Background

Lignocellulosic biomass has potential as an abundant and renewable feedstock for biofuel production. The main component of lignocellulosic biomass is the secondary walls of plant cells. A need for dedicated bioenergy crops with improved cell wall compositions and properties has become evident if biofuels are to be a cost-efficient alternative to fossil fuels [1,2].

Secondary cell walls of plants consist of cellulose microfibrils embedded in a matrix consisting mainly of hemicelluloses and lignin, the amounts of each single polymer varying widely between different species and cell types. Together these complex sugar polymers form rigid barriers that provide shape, structural strength and protection against environmental stresses and pathogens [2-4]. Hemicelluloses – mainly xylans – make up 20-35% of the secondary cell wall. In grasses, xylans are also the main noncellulosic polysaccharide in the primary walls [2,4,5]. As such, xylans represent a major part of the available biomass.

The bioconversion of lignocellulosic biomass into biofuels is currently suffering under the considerable recalcitrance of the biomass toward efficient deconstruction processes of the polymers into simple sugars [1,6]. Hemicelluloses and lignin embed cellulose microfibrils in a tight matrix, thus hindering access of cell wall degrading enzymes to the polysaccharides [7,8]. In addition, xylans are composed almost entirely of pentose sugars, which cannot be efficiently fermented [9,10]. Finally, xylans are heavily esterified with acetate, especially in hardwoods, and this hinders efficient enzymatic hydrolysis while the released acetate inhibits yeast fermentations [11,12]. For all these reasons, plants that have reduced amounts of xylan in their secondary cell walls, while still maintaining normal growth and development, would present a valuable feedstock for biofuel production.

Xylans are polysaccharides that have linear backbones of β-(1→4)-linked d-xylosyl residues that can be substituted with various side chains. The major xylan in dicot plants, glucuronoxylan (GX), is decorated with side chains of α-d-glucuronic acid (GlcA) and 4-*O*-methyl-α-d-glucuronic acid (MeGlcA). Moreover, acetylation is especially common in the secondary walls of this group of plants [5]. GXs from angiosperm and gymnosperm species have been shown to contain a reducing end oligosaccharide sequence consisting of β-d-Xyl*p*-(1→4)-β-d-Xyl*p*-(1→3)-α-l-Rha*p*-(1→2)-α-d-Gal*p*A-(1→4)-d-Xyl*p*[13-15]. The reducing end oligosaccharide has so far not been detected in grasses. It is not known whether this oligosaccharide functions as a primer for xylan biosynthesis or as a terminator sequence [15,16].

While the biosynthesis of other hemicelluloses with β-(1→4)-linked backbones involves the Cellulose Synthase Like (CSL) protein families, this does not appear to be the case for xylans [4,17]. Studies mostly of Arabidopsis mutants have led to the identification of several glycosyltransferases (GTs) with a role in xylan biosynthesis: IRX9/IRX9L [18] and IRX14/IRX14L [18,19] from GT family GT43 as well as IRX10/IRX10L [20,21] from GT47 seem to be involved in biosynthesis of the xylan backbone, while IRX8 (GAUT12) [15] and PARVUS (GATL1) [15,22] from GT8 and IRX7 (FRA8) and IRX7L (F8H) [23,24] from GT47 may be involved in synthesizing the reducing end oligosaccharide. All these GTs are predicted to be Golgi-localized type II membrane proteins. None of the proteins have had their biochemical activity determined and it is therefore not clear how they work together in the biosynthesis of xylan, but some studies indicate that they may function in protein complexes [25,26].

The GTs responsible for adding substitutions to xylan are better understood. Enzymes from GT61 are responsible for adding arabinosyl residues to the xylan backbone [27], and other enzymes from GT61 add xylose to such arabinosyl residues in grasses [28]. The glucuronosyl residues are added by GlucUronic acid substitution of Xylan (GUX) enzymes that belong to GT8 [29-31], and methyl groups are added to the glucuronic acid residues by a methyltransferase belonging to the DUF579 family [32].

The *irregular xylem* 7 (*irx7*), *irx8* and *irx9* T-DNA insertion mutants are deficient in GX biosynthesis, the resultant phenotype being the characteristic irregular xylems that arise when the weakened xylem vessels collapse inward, as they can no longer withstand the negative pressure that allows water to travel through the vessels. The *irx7*, *irx8*, and *irx9* Arabidopsis mutants have severely dwarfed whole plant morphologies and are largely infertile [33]. For both *IRX7* and *IRX9*, there are redundant genes, *IRX7L* and *IRX9L*, respectively, which are not highly expressed in tissues with secondary walls, but still influence the phenotype. The double knock-out mutations *irx7/irx7L* and *irx9/irx9L* are essentially lethal [18]. Different alleles have been described for *irx9* and the *irx9-2* mutant has a milder growth phenotype with a less severely stunted growth than the *irx9-1* mutant and remains fertile [15,18,34]. Through analyses of the *irx7*, *irx8* and *irx9* mutants, it was found that they are specifically expressed in developing vascular tissues where secondary walls are being deposited and are important for wall thickness and integrity. Xylose content in *irx7*, *irx8* and *irx9* is 28%, 35% and 45% less than wild type levels, respectively, and the mutants have significant reductions in cellulose content that is thought to be a consequence of the stunted growth of the plants and not a direct effect of the lost gene function. Together, these deficiencies cause large reductions in wall thickness of ~60% in all three mutants [15,33-35]. As the wall integrity is compromised in *irx7*, *irx8* and *irx9*, the mechanical breaking force of the stems is considerably lower than that of wild type plants [15,35]. In *irx7* and *irx8*, the reducing end oligosaccharide is largely absent, and they show a decrease in the number of GX chains. In contrast, the reducing end oligosaccharide is retained in the *irx9* mutant while the number of GX chains is increased and the chain length is decreased [15,34,35]. All these mutants retain substitution with MeGlcA but are devoid of GlcA substitution.

The spatial, temporal and quantitative expression of genes is controlled by transcription factors. The regulation of cell wall polysaccharide biosynthesis has been shown to involve a complex network of transcription factors, several of which are members of the plant specific NAC (NAM, ATAF1/2 and CUC2) domain proteins [36-38]. Vascular-related NAC Domain 6 (VND6) (At5g62380) and VND7 (At1g71930), together with the closely related NAC Secondary Wall Thickening Promoting Factor 1 (NST1) and NST3 (SND1) transcription factors, have been shown to be key regulatory switches for activation of secondary cell wall biosynthesis. VND6/VND7 and NST1/NST3 separately and/or collectively activate the biosynthetic pathways for cellulose, xylan and lignin through activation of a cascade of direct and indirect downstream transcription factors, many of which belong to the MYB family of transcription factors [39-42].These master regulators exhibit cell specific expression patterns, where VND6 and VND7 specifically regulate secondary cell wall biosynthesis in vessels while expression of NST1 and NST3 is confined to fibers [43-48]. *VND6* and *VND7* show vascular-specific expression patterns, the expression of *VND6* being specifically located in the inner-metaxylem vessels, while that of *VND7* is present in the protoxylem poles of the procambium region and in differentiating protoxylem and metaxylem vessels [43,49,50]. The function of *VND6* and *VND7* genes as key regulators of xylem vessel development was illustrated when they were overexpressed under the control of the cauliflower mosaic virus 35S promoter [43]. This experiment showed that various cell types could be transdifferentiated into xylem vessel elements with reticulated or pitted patterns like those of the metaxylem for the *VND6* construct, and into xylem vessels with annular or spiral patterns like those of protoxylem vessels for the *VND7* construct. Furthermore, dominant repression of either gene by fusion to the SRDX-domain specifically inhibited metaxylem and protoxylem formation, respectively [43].

**Figure 1:**
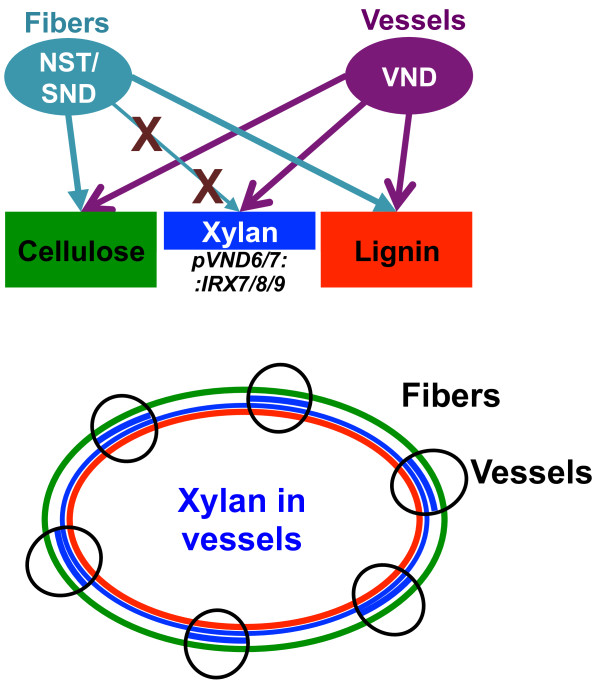
**Model of engineering strategy.** The xylan engineering strategy reintroduces xylan biosynthesis specifically into the xylem vessels of the xylan deficient *irx7, irx8* and *irx9* mutants. This is achieved through the expression of a functional allele of the defective *irx* gene under control of the vessel-specific *pVND6* or *pVND7* promoters.

The morphological effect of the *irx* mutations led us to hypothesize that the main reason for dwarfed growth in *irx* mutants is the collapsed vessels, and that specifically restoring xylan biosynthesis in vessels would therefore complement the mutations (Figure 1). We used Arabidopsis *irx7*, *irx8* and *irx9* mutants as backgrounds with strongly reduced amounts of GX in the secondary walls to reintroduce GX synthesis specifically in the vascular tissues, by exploiting the tissue-specific expression patterns of the *VND6* and *VND7* promoters. The dwarfed growth and *irx*-phenotype of the *irx7*, *irx8* and *irx9* mutants could be complemented to varying degrees, in some cases completely restoring wild-type growth patterns and mechanical properties while maintaining a low overall xylan content and improved saccharification properties.

## Results

### Vessel specific expression of the *IRX7*, *IRX8* and *IRX9* genes in the *irx7*, *irx8* and *irx9* mutant plants

**Figure 2:**
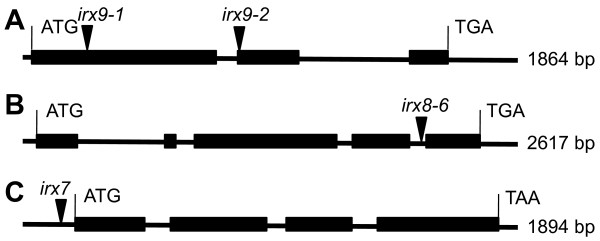
**Schematic illustration of the *IRX *genes and T-DNA insertions for the *irx *mutants.** (**A**) *irx9-2* in At2g37090 (*IRX9*) is located in the coding region of an exon. (**B**) *irx8-6* in At5g54690 (*IRX8*) is located in an intron. (**C**) *irx7* in At2g28110 (*IRX7*) is located in a non-coding region of an exon. T-DNAs are indicated with triangles.

**Figure 3:**
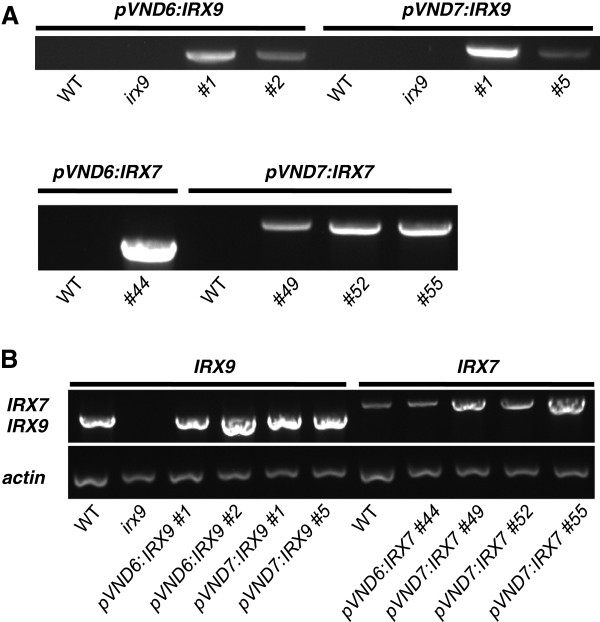
**Detection of transgene presence and expression.** (**A**) Agarose gels showing the presence of the transgene by PCR. Leaves from 6-week old plants were used. (**B**) RT-PCR analysis confirming expression of *IRX7* and *IRX9* genes respectively in stems of transformed plants. *Actin2* was amplified as control. cDNA was prepared from stems (top 5 cm) of 6-week old plants.

T-DNA insertional mutants in *IRX7*, *IRX8* and *IRX9* were used as genetic backgrounds for the xylan engineering (see Figure 2). The first attempt to engineer plants with decreased xylan content focused on the *irx9-2* (from here on referred to as *irx9*) mutant, which has the advantage of being fertile and transformable, in contrast to the *irx7* and *irx8-6* (from here on referred to as *irx8*) mutants. Thus, homozygous *irx9* plants were used for transformation with *Agrobacterium tumefaciens* (Agrobacterium) containing the pVND6:IRX9 and pVND7:IRX9 constructs. Due to the severely affected growth of the *irx7* and *irx8* mutants, plants homozygous for their respective defective *irx* allele have poor fertility [33,34]. For transformation of the *irx7* mutant with the pVND6:IRX7 and pVND7:IRX7 constructs, and of the *irx8* mutant with the pVND6:IRX8 and pVND7:IRX8 constructs, we selected plant lines that were heterozygous for the defective *irx7* and *irx8* allele, respectively. For each of the plant lines, positive T1 transformants were identified by screening for hygromycin resistance. Positive transformants in *irx7* and *irx8* backgrounds were also genotyped to identify hygromycin resistant plants that were homozygous for the defective *irx* allele. Plant lines with the best growth indicating good complementation of the *irx* phenotype were chosen for further analysis in T2. Prior to analysis of growth and cell wall properties, the selected plant lines were analyzed by PCR to confirm the presence of the respective transgene and by RT-PCR to ensure that the transgene was expressed (Figure 3).

### Growth complementation of rosettes and inflorescence stems

**Figure 4:**
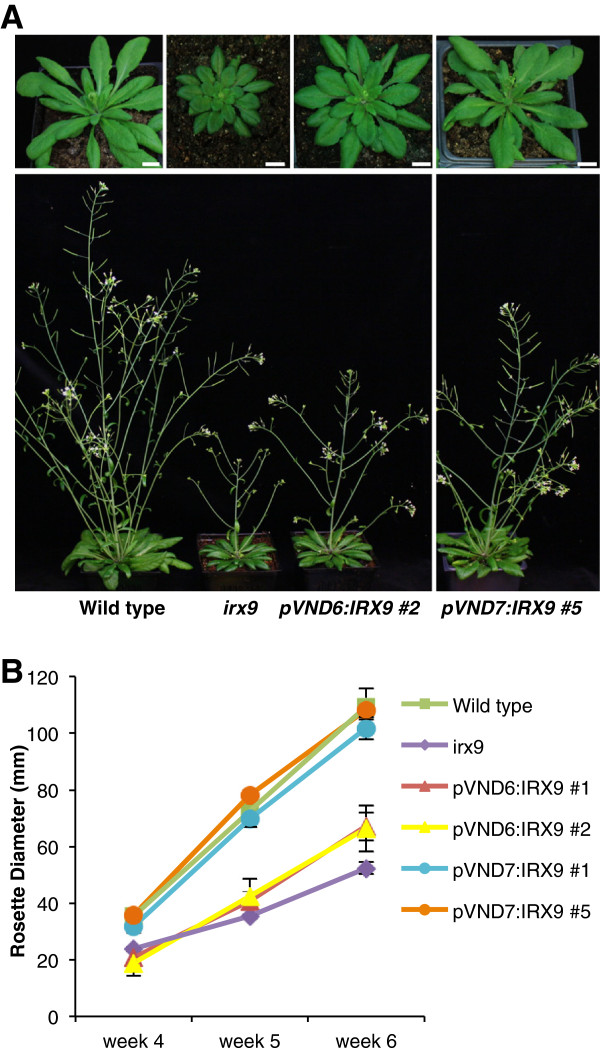
**Representative morphological phenotypes of plants showing growth complementation of the *irx9 *mutant.** (**A**) Scale bars: 10 mm. (**B**) The graph shows average diameter of the rosettes ± SE (n=5 for wild type, n=4 for *pVND6:IRX9* lines, n=7 for *pVND7:IRX9* #1, and n=6 for *pVND7:IRX9* #5).

**Figure 5:**
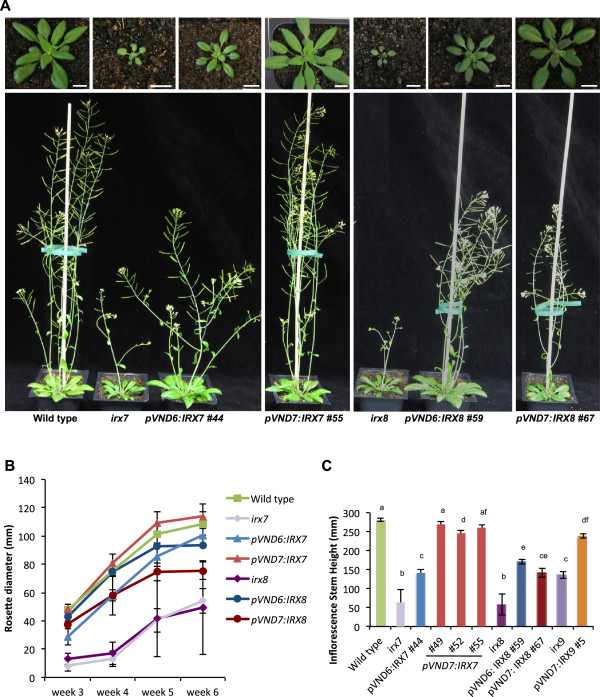
**Representative morphological phenotypes of plants showing growth complementation of the *irx7 *and *irx8 *mutants.** (**A**) Scale bars: 10 mm. (**B**) Average diameter of rosettes ± SE (n= 6); (**C**) Average height of the inflorescence stem ± SE (n=6). Bars that are not labeled with the same letter represent significantly different values (*t*-test, p < 0.05).

Weekly measurements of the growth of rosettes and inflorescence stems were conducted for the *irx* mutants transformed with the respective *pVND6* and *pVND7* constructs, along with the wild type and the *irx7, irx8*, and *irx9* mutants (Figures 4 and 5). The *irx7*, *irx8* and *irx9* mutants showed the characteristic dwarfed phenotype and smaller, dark green leaves previously described [33]. The morphology of *irx9* transformants containing the pVND7:IRX9 construct (Figure 4A and B) resembled that of the wild type at the rosette stage, but had a mildly stunted growth of the inflorescence stem compared to that of the wild type. Transformation with the pVND6:IRX9 construct (Figure 4A and B) yielded plants with a morphology that looked more similar to that of *irx9* mutants. The rosettes of pVND6:IRX7, pVND6:IRX8 and *pVND7:IRX8* (Figure 5A and B) transformants were smaller than those of the wild-type plants, while their inflorescence stems were intermediate in size between the wild type and their corresponding *irx* mutants (Figure 5A and C). In contrast, the *irx7* mutants harboring *pVND7:IRX7* (Figure 5) grew similarly to the wild type in respect to both rosette and stem growth, suggesting that the *irx* phenotype may be fully complemented in these transformants.

### Microscopic analysis of the *pVND6/pVND7:IRX7/IRX8/IRX9* expressing transformants

**Figure 6:**
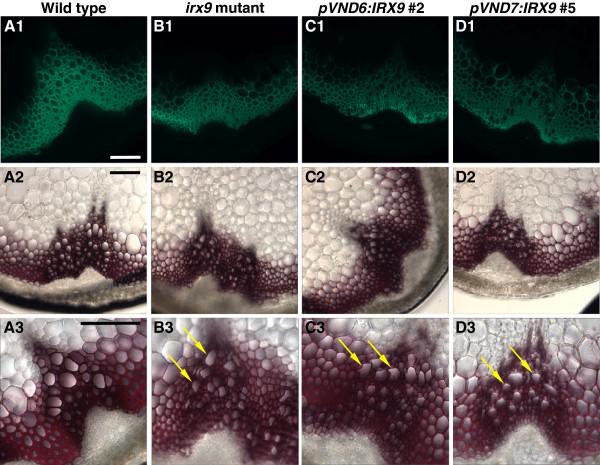
**Tissue analysis of stems in *irx9 *transformant lines.** (**A1**-**D1**) Immunodetection of xylan in transverse stem sections with the LM10 anti-xylan monoclonal antibody (**A2**-**D2**) Lignin deposition in the secondary walls was stained with Phloroglucinol-HCl. (**A3**-**D3**) Same as A2-D2, at higher magnification; arrows point to some of the irregular xylem cells. Scale bars for all panels: 100 μm.

**Figure 7:**
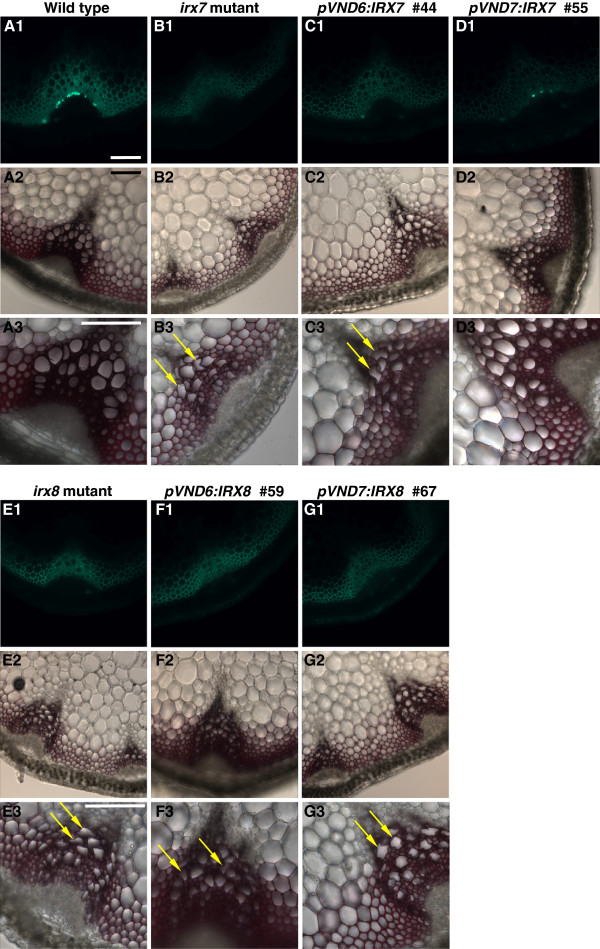
**Tissue analysis of stems in *irx7 *and *irx8 *transformant lines.** (**A1**-**G1**) Immunodetection of xylan in transverse stem sections with the LM10 anti-xylan monoclonal antibody. (**A2-G2**) Lignin deposition in the secondary walls was stained with Phloroglucinol-HCl. (**A3-G3**) Same as A2-G2, at higher magnification; arrows point to some of the irregular xylem cells. Scale bars for all panels: 100 μm.

The effect of the *pVND6/pVND7:IRX7/IRX8/IRX9* constructs on vascular tissue morphology was examined by analysis of transverse sections of the lower parts of inflorescence stems. Xylem vessels of wild type plants are characterized by large open cells that are relatively round in shape, while the interfascicular fibers are heavily lignified (Figure 6A) [51]. The xylem vessels of *irx7*, *irx8* and *irx9* are often smaller in size and have irregular shapes caused by large reductions in wall thickness (Figures 6B, 7B and E) [33].

Stem sections were immunolabeled with the LM10 monoclonal antibody to examine xylan distribution in the cell wall. The antibody recognizes unsubstituted and lowly substituted β-(1→4)-xylans [52] and its epitope has been shown to be specifically associated with cell types with secondary cell walls in Arabidopsis stems [53]. In the *irx* mutants, the decreased GX content resulted in a lower intensity of fluorescence in the xylem and interfascicular fibers when compared to the wild type sections.

Expression of *IRX9* under the control of the *pVND6* or *pVND7* promoter did not rescue the collapsed xylem vessels of the *irx9* mutant to a noticeable degree (Figure 6C and D). The pVND6:IRX7, pVND6:IRX8 and pVND7:IRX8 transformants had slightly less collapsed xylem cells compared to their respective *irx* mutant backgrounds, but still retained the thin cell walls in both xylem vessels and interfascicular fibers (Figure 7C, F, and G). In contrast, the pVND7:IRX7 transformants showed large open vessels that were comparable to those of the wild type (Figure 7D). The fluorescence intensity of the xylem vessels and the interfascicular fibers of pVND7:IRX7 plants resembled those of *irx7*, indicating reduced overall amounts of xylan compared to the wild type (Figure 7D).

The phloroglucinol-HCl stain was used to visualize lignin depositions in the cell walls [54]. Wild type stem sections showed thick lignified cell walls in both the vessels and interfascicular fibers. The *irx9* mutant contained wild type-like levels of lignin in the xylem vessels and reduced amounts in the interfascicular fibers (Figure 6B2-3). The relatively large degree of lignification in *irx9* may account for the less severe growth phenotype of this mutant. Lignin deposition in the pVND6/pVND7:IRX9 transformants was comparable to that of the wild type in the xylem vessels and to *irx9* in the interfascicular fibers, however the xylem vessels were still collapsed (Figure 6C2 and D2). In contrast, *irx7* and *irx8* mutant plants have very low levels of lignification in both the xylem vessels and interfascicular fibers (Figure 7B2-3 and E2-3). In the pVND6:IRX7 transformants lignin deposition was comparable to that of the *irx7* mutant (Figure 7C2). The pVND6:IRX8 transformants exhibited similar amounts of lignin to those of wild type in the xylem cells and in the interfascicular fibers (Figure 7F2-3), while lignification in the pVND7:IRX8 transformants was intermediate between that of *irx8* and wild type in both cell types (Figure 7G2-3). In the pVND7:IRX7 lines lignification was almost completely restored in both xylem cells and interfascicular fibers (Figure 7D2-3).

### Analysis of cell wall composition

**Figure 8:**
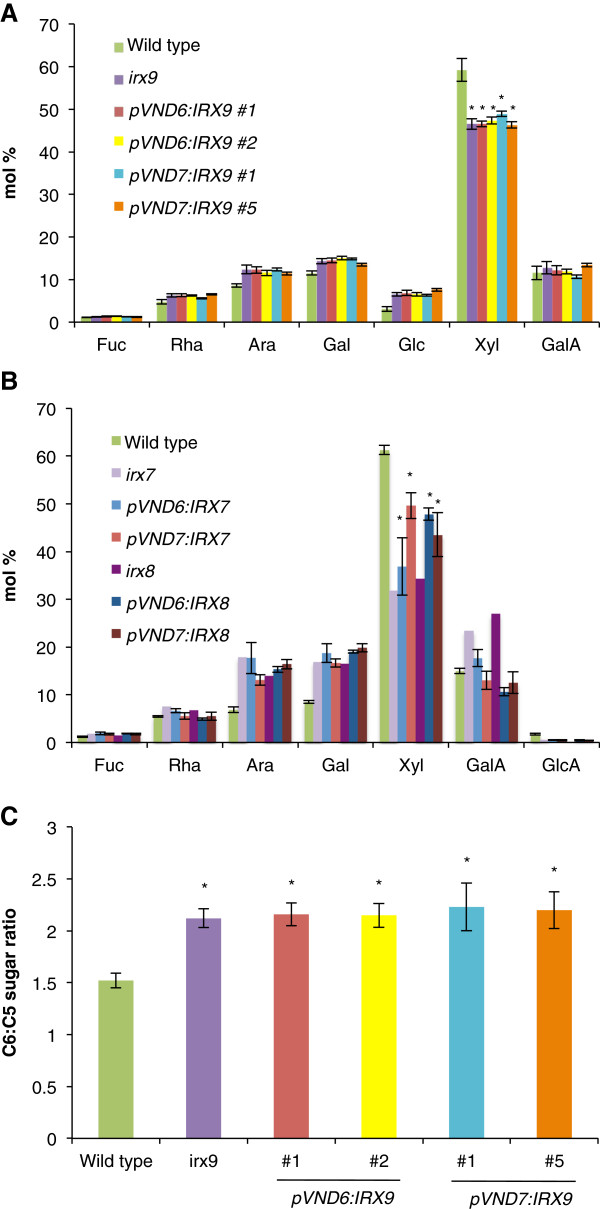
**Monosaccharide composition of the cell wall from *irx9, irx7 *and *irx8 *plants transformed with *IRX9*, *IRX7 *and *IRX8 *under the native *VND6 *and *VND7 *promoter.** Cell wall material (alcohol insoluble residue, AIR) was prepared from fresh stem material, hydrolyzed with trifluoroacetic acid (A and B) or sulfuric acid (C), and analyzed by high-performance anion exchange chromatography. (**A**) All *irx9* transformant lines have significantly reduced xylose content that are similar to those of the *irx9* mutant. (**B**) The *irx7* and *irx8* transformant lines have intermediary xylose levels compared to the wild type and their respective *irx7/irx8* backgrounds. (**C**) Hexose/pentose ratio in cell walls of transformed *irx9* plants. The sugar content in the cell walls was determined after complete hydrolysis with sulfuric acid. All graphs show average ± SD (where (**A**) and (**B**) n=4 and (**C**) n=3) except for *irx7* and *irx8* mutants where only one replicate was available. * indicates a significant difference in xylose or C6/C5 ratio from the wild type (*t*-test, p < 0.01).

Monosaccharide compositional analysis after hydrolysis with TFA was performed on cell wall preparations from the basal part of the inflorescence stem. This analysis demonstrated that xylose contents in all transformants were reduced to levels resembling that of the respective *irx* mutant or to intermediate levels between that of the mutant and the wild type (Figure 8A and B). Cell wall monosaccharide analysis of the *irx9* mutant transformed with the pVND7:IRX9 construct (Figure 8A) revealed a significant 17% reduction in xylose content compared to the wild type, while the *irx7* transformants containing the pVND7:IRX7 construct (Figure 7B) showed significant reductions between 16-23% compared to the wild type. An overall increase for all other monosaccharides could be observed corresponding to the decrease in xylose.

Transformants in the *irx9* background were additionally subjected to analysis of total wall monosaccharide composition after hydrolysis with sulfuric acid, in order to calculate the ratio between total C6 and C5 sugars in the wall (Figure 8C). Transformants expressing the pVND6:IRX9 or the pVND7:IRX9 constructs exhibited a ratio of C6/C5 sugars that was increased up to 46% compared to the wild type, which is a ratio similar to that of the *irx9* mutant.

### Stem breaking-force measurements

**Figure 9:**
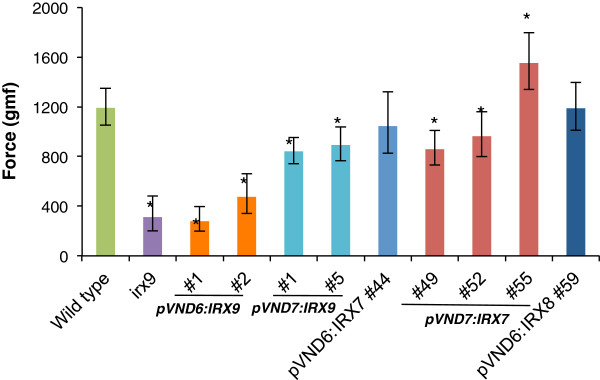
**Breaking-force measurements of inflorescence stems.** The tensile breaking strength of the main inflorescence stem of 7-week-old plants was tested. Values show average ± SD (n=5-9) of log-transformed data. * indicates a significant difference from the wild type (*t*-test, p < 0.05).

To test the physical properties of the transformed plants, we performed breaking-force measurements on stem sections of 7-week-old plants (Figure 9). As expected, the *irx9* mutant had a strongly reduced tensile strength compared to the wild type, and transformation with the pVND7:IRX9 constructs only partially restored the stem strength while transformants with the pVND6:IRX9 constructs were as weak as the *irx9* mutant. Transformants of the *irx7* mutant all showed significant recovery of stem strength. For the *irx7* mutant transformed with the pVND6:IRX7 construct (line #44) stem strength was the same as in the wild type, and in line #55 with the *pVND7:IRX7* construct stem strength even exceeded that of the wild type by 30% (Figure 9). The *irx8* mutant transformed with the pVND6:IRX8 constructs also exhibited stem strength comparable to the wild type. Generally, the degree of recovery of stem strength was consistent with the recovery of growth and stem morphology discussed above (Figures 4, 5, 6, and 7).

### Quantification of lignin content

**Figure 10:**
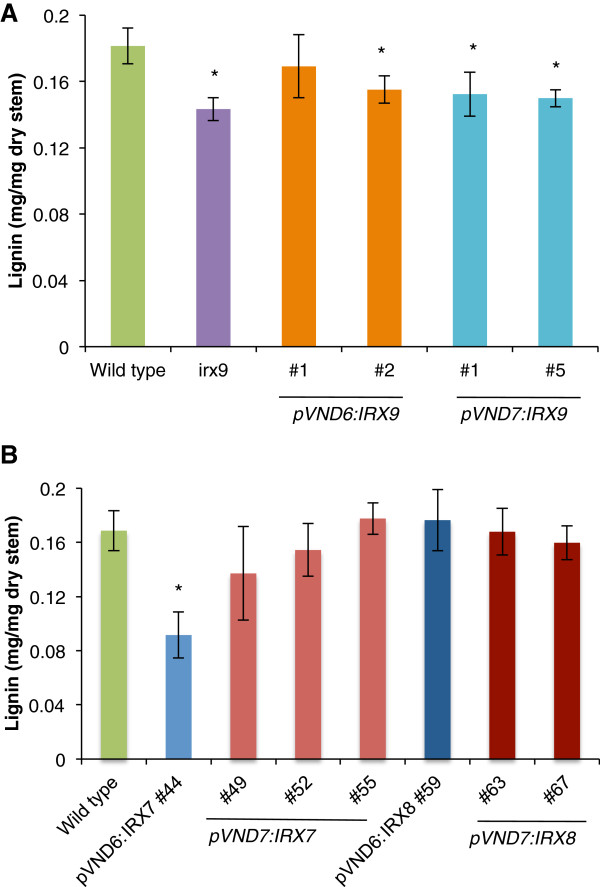
**Lignin quantification.** (**A**) *irx9* transformed lines. (n=3) (**B**) *irx7* and *irx8* transformed lines (n=4). Dry material from senesced stems was used for this analysis. Values show average ± SD. * indicates a significant difference from the wild type (*t*-test, p < 0.05).

Due to the reduction in lignin content observed with the phloroglucinol-HCl staining method of inflorescence stem sections, we quantified the content of lignin in the transformed plants with the acetyl bromide assay. Transformants of *irx9* harboring the *pVND6/*pVND7:IRX9 constructs showed a 14-17% reduction in lignin content compared to the wild type (Figure 10A). Transformants in the *irx7* background harboring the pVND6:IRX7 construct had a significantly lower lignin content compared to the wild type, whereas the other transformants in *irx7* and *irx8* mutant backgrounds did not show significant changes in lignin content (Figure 10B).

### Improvement in sugar release after saccharification

**Figure 11:**
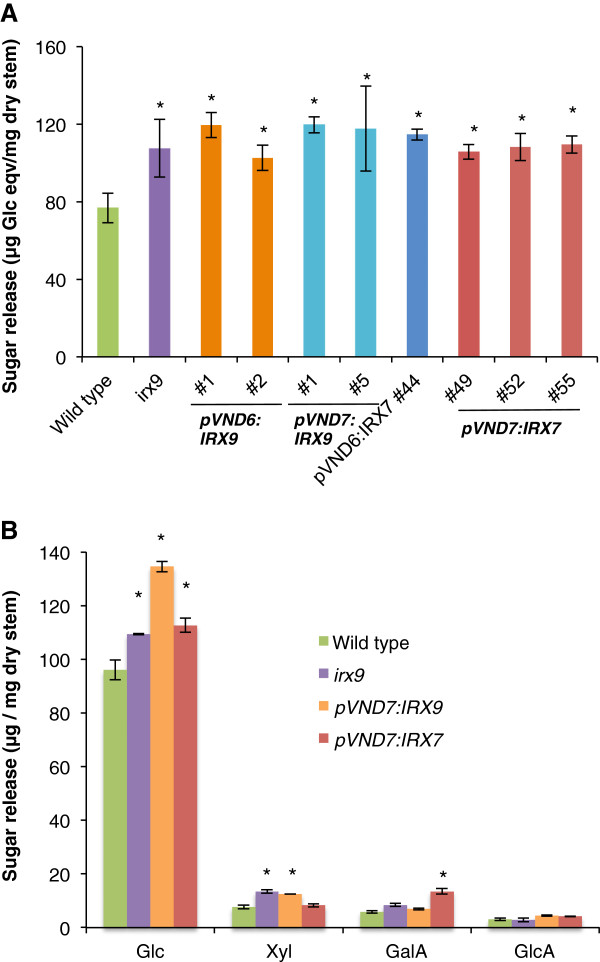
**Saccharification analysis.** Hot water pretreatment of dry stem material was followed by 24 h of saccharification with the Cellic CTec2 (Novozymes) enzyme mixture. (**A**) Release of reducing sugar was determined by the dinitrosalicylic acid assay. All transformants showed increases in saccharification yield compared to the wild type. Values show average ± SD (n=7 for wild type, *irx9* mutant and *pVND7:IRX9* #5, both *pVND6:IRX9* lines and *pVND7:IRX9* #1 n=3, and all remaining lines n=4). (**B**) Released sugars from selected lines were analyzed by HPAEC. Values show average ± SE (n = 3 for wild type and *irx9,* and n=8 for *pVND7:IRX9* and *pVND7:IRX7* lines). Fucose, rhamnose, arabinose and galactose were present in trace amounts. * indicates a significant difference from the wild type (*t*-test, p < 0.05).

Upon hot water pretreatment and after 24 h of enzyme digestion with the Cellic CTec2 enzyme cocktail, all *irx* transformant lines showed an increase in saccharification yield compared to that of the wild type (Figure 11A). The *pVND6/*pVND7:IRX9x lines showed improved saccharification yields of up to 55%, while the pVND6:IRX7 and pVND7:IRX7 lines showed improved yields of up to 49% and 42%, respectively. The released sugars from the two pVND7:IRX9 and three pVND7:IRX7 lines were further analyzed by HPAEC (Figure 11B). Clearly, the Cellic CTec2 enzyme mixture was most efficient in releasing glucose, with a much less efficient release of xylose. The *irx9* mutant and the transformants all showed an increased release in glucose. However, it is not possible to conclude if this increase is due to better accessibility to cellulose or simply reflects that the biomass has relatively higher cellulose content. The *irx9* mutant and pVND7:IRX9 lines also showed increased release of xylose, which is somewhat surprising given that these lines have lower xylan content. This suggests that xylan is more accessible to enzymatic breakdown in these lines and may be explained by their lower lignin content (Figure 10B). In contrast, the pVND7:IRX7 lines which did not show an increased xylan release (Figure 11B) had normal lignin content (Figure 10B).

## Discussion

### Complementation of the *irx7, irx8* and *irx9* growth phenotypes through vessel-specific expression of *IRX7, IRX8,* and *IRX9*

The *irx7*, *irx8* and *irx9* mutants have large reductions in GX content, a trait that increases saccharification yields considerably, but also reduces total biomass yields through severely impaired growth of the plants [7,8,33]. The phenotype of these and other mutants affected in xylan biosynthesis has demonstrated that xylans are essential polysaccharides in plant cell walls. However, the results presented here show that while xylans are essential for plants, they are not essential in all cell types. Indeed, the stunted growth of the *irx* mutants appears to be caused predominantly by collapsed xylem vessels and subsequent impaired nutrient and water transport. Consequently, we could alleviate the growth phenotype and still retain the low xylan levels of the *irx* mutants by an engineering approach that enables the re-introduction of xylan biosynthesis specifically in the xylem vessels of xylan deficient *irx* mutants.

Expression of the wild type *IRX7*, *IRX8* and *IRX9* cDNAs under control of the *pVND6* or *pVND7* promoter in the respective *irx7*, *irx8* and *irx9* mutant backgrounds yielded improved growth patterns of both rosette size and stem height for all transformant lines, when compared to the respective *irx* mutant (Figures 4 and 5). As expected, individual lines showed variation in the degree of growth complementation. However, overall there were some general patterns found. For both the *irx7* and *irx9* mutant backgrounds, complementation with the *pVND7* promoter worked better than the *pVND6* promoter (Figures 4 and 5). This difference must be due to the differences in the expression profiles of these promoters. Since both pVND6:IRX9 and pVND7:IRX9 lines show the same low xylan content in the walls, it is not because the *pVND7* promoter is necessarily stronger than *pVND6*. The *VND6* transcription factor gene is specifically expressed in metaxylem vessels, while *VND7* has been shown to be expressed in both the protoxylem and metaxylem vessels [43,49]. The broader expression pattern when expressing xylan biosynthetic genes under the *VND7* promoter may account for the better growth complementation in these plants. Expression of xylan in the metaxylem only, with the pVND6:IRX7/IRX8/IRX9 constructs might not be sufficient to fully restore the irregular xylems of the *irx* mutants. This result contrasts with our recent study where the same *pVND6* promoter construct was used to complement a mutant in lignin biosynthesis [50]. In that study, the pVND6:C4H construct fully complemented the irregular xylem phenotype of the *c4h* mutant. Such difference could be explained by the capability of the monolignols to diffuse in the cell wall prior to being polymerized into lignin in contrast to xylan polymers.

Irrespective of the promoter, complementation of *irx7* worked better than *irx9*, which in turn worked better than *irx8*. The reason for this is not clear and it is difficult to speculate about it given the lack of knowledge about the biochemical function of the IRX7, IRX8 and IRX9 proteins. The three pVND7:IRX7 transformant lines all showed growth very similar to the wild type. The partial growth complementation seen for many of the other transformants was reflected in the still collapsed xylem vessels in these plants (Figures 6 and 7). The only transformants that showed the large open vessels characteristic for the wild type were the ones expressing the pVND7:IRX7 construct (Figure 7D1-3). In stem sections from the pVND7:IRX7 transformants, the fluorescence intensity of LM10 was similar to that of *irx7* indicating that xylan levels were low, while the interfascicular fiber cell walls remained thin compared to the wild type. This result shows that it is possible to obtain plants that have reduced amounts of xylan in their walls, while still preserving the structural integrity of the xylem vessels.

### Transformants expressing the *pVND6/pVND7:IRX7/IRX8/IRX9* constructs showed decreased levels of xylan and lignin compared to wild type

The *irx7*, *irx8* and *irx9* mutants that were used as backgrounds for the transformations are xylan-deficient [33]. By expression of xylan biosynthetic genes specifically in the xylem vessels of these mutants, it was possible to rescue the strong growth phenotypes of the *irx* mutants, while all the transformed mutants still showed large decreases in xylose compared to the wild type, to levels resembling that of the respective *irx* mutant (Figure 8A) or to an intermediate level between the mutant and the wild type (Figure 8B). The three pVND7:IRX7 transformant lines with growth properties similar to wild type, had xylose contents that were 16-23% lower compared to the wild type.

Reductions in lignin has been proven to be important for decreasing the recalcitrance of biomass toward deconstruction, making the reductions in lignification observed here promising for increasing saccharification yields. The pVND6:IRX9 and pVND7:IRX9 transformants had reductions in lignin content to levels similar to that of the *irx9* mutant (Figure 10A), while the transformants expressing the pVND6:IRX7 or pVND7:IRX7 construct had near wild type levels of lignin deposition (Figure 10B). That lignin deposition is affected in the complemented plants suggests that the degree of lignification of vascular tissues is dependent on xylan biosynthesis. There are several possible explanations for the decrease in lignin conferred by the loss of xylan. Since the transformants have an overall decrease in secondary wall development, the reduction in lignin may simply reflect that. However, the branches of GX polymers in Arabidopsis consist of GlcA and MeGlcA, and it is possible that the GlcA and MeGlcA substitutions have a function in interacting with lignin polymers by covalent attachment through esters [30]. Hence, there may be a more specific effect of the xylan reduction on lignin accumulation in the walls.

### Transformants showed improved stem breaking strength

Wall integrity has been demonstrated to be compromised in the *irx7*, *irx8* and *irx9* mutants, causing a decrease in the breaking strength of the inflorescence stems [15,35]. It is essential that plants bred or engineered for improved downstream processing do not have impaired growth or susceptibility e.g. to lodging. In general, the transformed *irx* mutants showed partial recovery of stem strength and several lines were as strong as the wild type. One of the transformant lines expressing the pVND7:IRX7 construct in the *irx7* background was even 30% stronger than the wild type. Thus, it is possible to obtain plants that are not compromised in mechanical properties using the strategy described here.

### The decrease in xylan and lignin content in the secondary walls in the transformants results in improved saccharification and a more optimal C6/C5 sugar ratio of hydrolyzates

The lower amounts of xylose and lignin observed in the secondary walls of the transformants were expected to yield higher saccharification efficiencies, as these two polymers are known to be the main contributors to cell wall recalcitrance [7]. We saw large increases in saccharification yields in all transformants compared to the wild type of up to 49% and a wild type like growth phenotype for the lines complemented with the pVND7:IRX7 construct. As inefficient enzymatic degradation of plant biomass is one of the major bottlenecks in achieving economically feasible biofuel production, the xylan engineering system we presented here is a great step toward tailored bioenergy crops that can alleviate the problem of biomass recalcitrance toward degradation. Furthermore, the hydrolyzates obtained by complete saccharification of the transformants have C6/C5 sugar ratios that are increased by up to 46% compared to the wild type.

The economic impact of improved feedstocks properties as reported in this paper is difficult to assess without pilot plant experiments. However, a very thorough techno-economical modeling of biomass to ethanol conversion allows good estimates to be made [55]. According to this model, a 20% decrease in xylose and 10% decrease in lignin content would result in about 10-15% decrease in minimum ethanol selling price, provided that plant growth would not be negatively impacted.

### Engineering of bioenergy crops

The engineering approach described here was done in the model plant Arabidopsis. The future of tailored plants for biofuel production has been proposed to lie within fast growing plant species with high biomass yields. Fast growing C4 perennial grass species, such as Miscanthus (*Miscanthus distachyon*) and Switchgrass (*Panicum virgatum*) are promising as future bioenergy crop species, while hybrid poplar (e.g. *Populus alba* x *tremula*) show potential as a woody energy crop [6].

Several functional orthologs of the *irx* genes involved in xylan biosynthesis in Arabidopsis have been identified in hybrid poplar [56]. Here, *GT47C*[57] and *GT8E/F*[58] may function in the biosynthesis of the reducing end oligosaccharide of GX, as they are functional orthologs of *IRX7/F8H* and *PARVUS*, respectively, in Arabidopsis. *GT8D* is an ortholog of Arabidopsis *IRX8* and has been shown to be important for maintaining the mechanical strength and xylan content in poplar, while *GT43B* has been shown to be a functional ortholog of Arabidopsis *IRX9*[59,60]. The approach described in this paper depended on pre-existing mutants in *irx* genes, and such mutants may not be readily available in crop species. However, *GT43B* RNAi lines have been reported to have alterations in xylan content and xylan chain length that yield a reduction in recalcitrance to cellulose digestion [56]. These findings suggest that it will be possible to transfer our xylan engineering approach to poplar species in the near future by retransformation of plants where the native gene expression has been suppressed (e.g. by RNAi) with an RNAi-insensitive *IRX* allele driven by a vessel-specific promoter from poplar [61]. In grasses the situation is less clear, since the reducing end oligosaccharide of xylan has not been identified and it is unknown if there are functional orthologs of *IRX7*, *IRX8* and *PARVUS*. However, grasses do have apparent orthologs of *IRX9* and *IRX9L* and they could be targeted in a similar way through RNAi and transformation with a functional copy of *IRX9* under control of a vessel-specific promoter. Orthologs of *IRX10,* which appears to be present in grasses [25], may be targeted in a similar way.

Transfer of the engineering approach to crop species, also requires vessel-specific promoters. Phylogenetic analysis suggests that VND6 and VND7 are highly conserved, and we expect that the Arabidopsis promoters will function in a wide range of species. If the expression level is too low to fully complement growth, extra copies of *VND7* under control of its native promoter could be introduced. The findings, that Arabidopsis and poplar share many of the same biosynthetic networks [62,63] sets the basis for introducing the transcription factor mediated xylan biosynthesis in poplar, the same way we have now demonstrated for Arabidopsis.

## Conclusions

During this study, we have shown that it is possible to apply a xylan engineering approach by manipulating the spatial and temporal deposition of this abundant polymer specifically to the xylem vessels in mutant Arabidopsis plants deficient in GX biosynthesis. We obtained Arabidopsis plants that have up to a 23% reduction in xylose levels compared to wild type plants. These transformants exhibit wild type-like growth patterns and morphology and normal xylem vessels. Furthermore, these plants showed up to a 42% increase in saccharification yield after hot water pretreatment and 24 h of incubation with an enzyme mixture. The best results were obtained by transforming *irx7* mutants with the pVND7:IRX7 construct. Several other plant lines in the *irx9* and *irx8* backgrounds yielded partial complementation of the *irx* phenotype thus showing promise of improvement in future experiments. The breaking-force tests show that the improvement in xylose-reduction and lignin content can be achieved without compromising mechanical strength of the plants.

The xylan engineering system developed in this study has the potential of being transferred to other biofuel crop species. Particularly poplar species have been shown to contain functional orthologs of the Arabidopsis *IRX* genes, and for the biosynthesis of cell wall polymers to be regulated by a transcriptional regulatory system similar to that in Arabidopsis.

## Methods

### Plant lines and growth conditions

All Arabidopsis wild type and mutant plant lines used are in the ecotype Columbia (Col-0) background. T-DNA insertion mutants (*irx9-2*, At2g37090, SALK_057033; *irx7*, At2g28110 SALK_120296; *irx8-6*, At5g54690, SALK_008642, [64]) were obtained from the Arabidopsis Biological Resource Center, Ohio State University, (http://www.arabidopsis.org). Wild type and T-DNA insertion mutant seeds were grown on soil at 22°C in a 16 h photoperiod after being stratified at 4°C for 4 days. Following transformation, seeds were harvested, sterilized and then grown on plates containing MS media (0.5x Murashige and Skoog salts, 7 g/l agar, 10 g/l sucrose) with 30 μg/ml hygromycin selection and stratified for 4 days at 4°C. Plates were then transferred to growth chambers at 22°C with 10 h photoperiod for 7–10 days. Positive transformant seedlings were transferred to soil.

Transformants in the *irx9* background (except plants used for stem tests, expression analysis, and transgene screening, which were grown entirely with a 16 h photoperiod) were grown for 4 weeks at 22°C in 10 h photoperiod and then moved to 16 h photoperiod. Transformants in the *irx7* and *irx8* backgrounds were grown entirely with a 16 h photoperiod.

### Vector constructs and Arabidopsis transformation

The native *VND6* promoter containing a 2757 bp region upstream of the ATG codon was amplified from genomic DNA using the following primers, pVND6-F3-KpnI, cccgggtaccTCCTTTACGATGTTGTTATGGGTTA; pVND6-R3-SpeI, cccgactagtGTGTGCGAGA CTTTGGATTTGAT CTTTTTAATTTTA [50]. The native *VND7* promoter containing a 2009 bp region upstream of the ATG codon was amplified from genomic DNA using gene specific primers pVND7NotI-5, CCCGGCGGCCGCTTCTGTAGTTCTTCTTCGGGTTTACAAATC; pVND7-NheI-3, CCCGGCTAGCATTATCCATCCACGATGATCCTATAAACGT. The PCR products were cloned into pBlunt (Invitrogen, Carlsbad, CA) to create pBlunt-p*VND6* and pBlunt-p*VND7* respectively. A Gateway cloning cassette was inserted between HindIII and AvrII restriction sites of the binary vector pCAMBIA 1390 (accession no. AF234307) to produce a pA6-GW vector. The promoter of *VND6* was cut from pBlunt-pVND6 by KpnI and SpeI, and then inserted between KpnI and AvrII (SpeI compatible) restriction sites of the binary vector pA6-GW to produce the pA6-p*VND6*-GW vector. The promoter of *VND7* was obtained by restriction digestion with KpnI and NheI from pBlunt-p*VND7* and then inserted in the binary vector pA6-GW vector to produce the pA6-p*VND7*-GW vector.

The full-length coding regions of At2g28110 (*IRX7*), At5g54690 (*IRX8*) and At2g37090 (*IRX9*) were PCR amplified from Arabidopsis cDNA and cloned into pDONR/Zeo (Invitrogen) (for *IRX7*) and pCR8/GW/TOPO (Invitrogen) (for *IRX8* and *IRX9*) by Gateway BP and Gateway TOPO reaction, respectively. LR reactions were set up using these entry vectors to clone the coding region into the Gateway compatible destination vectors pA6-*pVND6*-GW and pA6-*pVND7*-GW. After verification by sequencing, all constructs were transformed into Agrobacterium strain GV3101.

Homozygous *irx9* mutants and heterozygous *irx7* and *irx8* plants were used for transformation with the floral dip method [65] for each of the above constructs, respectively. T1 seeds were sown on hygromycin plates and positive transformants were transferred to soil followed by subsequent verification of the genotype using PCR.

### Screening of transformants for T-DNA insertions and transgenes

Identification of the positive transformants that were homozygous for the T-DNA insertions resulting in the *irx9-2*, *irx7*, and *irx8-6* mutants were done as described by [33]. Primer sets of right- and left-border primers (RP+LP) specific for each T-DNA insertion were generated from the SIGnAL T-DNA Primer Design website (http://signal.salk.edu/tdnaprimers.2.html) as shown in Table 1.

**Table 1:** Plant lines and primers used for genotyping

**Mutant Line**	**Gene Locus**	**Insertion line**	**Primer sequence**
*irx7*	At2g28110	SALK_120296	5’-CGTCATTGATGAGTGATCGTG-3’ fwd
			5’- TGCAAAATTTGTCATTGTCAC-3’ rev
*irx8-6*	At5g54690	SALK_008642	5’-TCAAGAAAAATCATCAACCGG-3’fwd
			5’-AACAGCATTAGACCCGAAACC-3’ rev
*irx9-2*	At2g37090	SALK_057033	5’- GCTGGTAAGGCCTCATTTTTC-3’ fwd
			5’- AACTTACCAACCCACCCATTC-3’ rev

The primers were used to identify transformants that were homozygous for the T-DNA insertions yielding the *irx9-2*, *irx7* and *irx8* mutants. Primer sets of right- and left-border primers (RP+LP) specific for each T-DNA insertion were generated from the SIGnAL T-DNA Primer Design website (http://signal.salk.edu/tdnaprimers.2.html). T-DNA left border primers used were LBa1 (5’-TGGTTCACGTAGTGGGCCATCG-3’) and LBb1.3 (5’-ATTTTGCCGATTTCGGAAC-3’).

To confirm the presence the transgene in the transformants, primer pairs were made for the respective *pVND* promoter and *IRX* gene combination and used in PCR reactions. Primer sequences used are shown in Table 2.

**Table 2:** Primers used to confirm presence and expression of the transgenes

**Primer name**	**Primer sequence**
pVND6_irx7/9_FW	5’-GAATCCACCATCATCGGAGT-3’
pVND6_irx7_RV	5’-TCGGCTTCATACGGATCTTC-3’
pVND6_irx9_RV	5’-CCACAATAAGGGAGGTGGAA-3’
pVND7_irx7/9_FW	5’-ATGCACGAACCAGATGATGA-3’
pVND7_irx7_RV	5’-CGAGAAGCGAAAAGCTTAGG-3’
pVND7_irx9_RV	5’-TCAAAAGGTTGGGGAGGAAT-3’
IRX7_RT-PCR_FW	5’-ATGACAACACATAAACATAGAAGAAC-3’
IRX7_RT-PCR_RV	5’-CAAGAAAGAGTTTGACCTTCTAACA-3’
IRX9_RT-PCR_FW	5’-ATGGGATCTCTAGAGAGATCA-3’
IRX9_RT-PCR_RV	5’-GGTGCTTAAACGTGTTCTTGTG-3’
ACT2_FW	5’-CTCAAA GACCAGCTCTTCCATC-3’
ACT2_RV	5’-GCCTTTGATCTTGAGAGCTTAG-3’.

### Transcript analysis of transformants

RNA was isolated from stems using Qiagen RNeasy plant mini kit. First-strand cDNA synthesis was done using SuperScript II RT (Invitrogen). For the PCR steps, full-length gene-specific primer pairs were used. Primer sequences are shown in Table 2. (*IRX7* forward 5’-ATGACAACACATAAACATAGAAGAAC-3’ and reverse 5’-CAAGAAAGAGTTTGACCTTCTAACA-3’; *IRX9* forward 5’-ATGGGATCTCTAGAGAGATCA-3’ and reverse 5’-GGTGCTTAAACGTGTTCTTGTG-3’). *ACTIN2* control was amplified using *ACT2*-fwd 5’-CTCAAA GACCAGCTCTTCCATC-3’ and *ACT2*-rev 5’-GCCTTTGATCTTGAGAGCTTAG-3’.

### Monosaccharide composition analysis

Alcohol-insoluble residue (AIR) of inflorescence stems was prepared and destarched enzymatically as previously described [66]. For the *irx7* and *irx8* transformant lines, AIR was not destarched. All AIR samples were subsequently hydrolyzed with 2 M triflouroacetic acid (TFA) for 1 h at 120°C. For the *irx9* transformant lines, completely dried senesced stem material was furthermore subjected to total sugar hydrolysis using the sulfuric acid method [67]. Samples were treated with 50 μl 72% w/w sulfuric acid and incubated for 1 h at 30°C while shaking. Samples were diluted with 715 μl of water and incubated at 120°C for 1 h. Sulfuric acid hydrolyzed samples were diluted 200-fold and the monosaccharide composition was determined by high-performance anion exchange chromatography (HPAEC) using a Dionex 3000 ion chromatograph as described [66].

### Preparation of stem material for analysis by microscopy

The base of 6 week-old main inflorescence stems (2.5 cm distal from the rosette) were harvested and fixed overnight in fixative solution (4% paraformaldehyde in 50 mM piperazine-N,N^′^-bis(2-ethanesulphonic acid) and 5 mM EGTA, pH 6.9) at 4°C as described in [68]. The stems were embedded in 7% agarose and cut into 60 μm thick sections using a Leica VT1000S vibratome and used for all subsequent microscopy analyses as described in [53].

### Immunofluorescent labeling for light microscopy

Immunofluorescent labeling of transverse stem sections was done using the xylan-specific rat monoclonal antibody LM10 (Plant Probes) [52]. Sections were labeled as described in [68] with minor deviations. Sections were incubated with the primary antibody diluted 10-fold in a milk powder protein solution (5%, w/v) in phosphate buffered saline (PBS) for 1.5 h at room temperature. After extensive washes in PBS, sections were incubated in the dark with a secondary antibody (anti-rat/FITC) diluted 100-fold in PBS with 5% milk powder for 1.5 h at room temperature and washed again extensively with PBS. Pictures were taken using a Micropublisher Q-imaging camera (5.0 RTV) mounted on an epifluorescent microscope (Leica DM4000B) coupled with Metamorph software.

### Force measurements of stem breaking force

Sections of main inflorescence stems from 100 to 150 mm above the rosette were taken from 7 week-old plants and the ultimate breaking force was measured using an in-house tensile testing instrument described previously [69].

### Phloroglucinol-HCl staining of lignin

Fixed stem cross sections from each transformant line were treated one at a time with 2% phloroglucinol (Sigma, P3502) in a 2:1 ethanol/HCl solution [54]. The sections were analyzed on a Leica DM4000B microscope through the Bright-field filter.

### Acetyl bromide assay for lignin quantification

Lignin quantification was determined by the acetyl bromide spectrophotometric method with some modifications [70,71]. Acetyl bromide solution (600 μl of 25% v/v acetyl bromide in glacial acetic acid) was added to ~5 mg of AIR samples and incubated for 3 h at 50°C while shaking. Samples were cooled on ice to room temperature and centrifuged at 18407 g for 5 min. Subsequently, 100 μl of sample was transferred to a new tube. 400 μl of 2 M NaOH and 70 μl 0.5 M hydroxylamine hydrochloride was added followed by vortexing. 57 μl of each sample were transferred to a UV-transparent 96 well plate, filled up to 200 μl with glacial acetic acid and the samples analyzed in a plate reader at 280 nm.

### Hot water pretreatment and saccharification

Cell wall pretreatment and saccharification was determined following a modified procedure from [54]. Dried, senesced stem material (5 mg fine powder) was pretreated with 200 μl of water and then incubated with shaking for 30 min at 30°C, followed by incubation for 1 h at 120°C. The samples were then allowed to cool to room temperature. For enzymatic saccharification, a mixture of 5 mg/ml tetracycline and Cellic CTec2 enzyme mix (Novozymes, Bagsværd, Denmark) in 0.1 M citrate buffer, pH 5.0 was added to the pretreated samples, followed by incubation at 50°C for 24 h at 900 rpm. Following addition of dinitrosalicylic acid reagent (1 g dinitrosalicylic acid and 30 g potassium sodium tartrate in 100 ml of 0.4 M NaOH) to the samples, aliquots were extracted and analyzed at the beginning of the experiment (T=0) and after 24 h (T=24). Samples were read on a plate reader at 540 nm. In addition, the released monosaccharides were analyzed by HPAEC as described above.

## Abbreviations

CSL: Cellulose synthase like; GlcA: α-D-glucuronic acid; GUX: GlucUronic acid substitution of Xylan; GX: Glucuronoxylan; GT: Glycosyltransferase; *Irx*: Irregular xylem; MeGlcA: 4-*O*-methyl-α-D-glucuronic acid; NAC: NAM ATAF1/2 and CUC2; NST: NAC secondary wall thickening promoting factor; VND: Vascular-related NAC domain.

## Competing interests

The strategy described in this paper has been included in a patent application.

## Authors’ contributions

DL and HVS designed and coordinated the study; HVS, PDP, JL, BE, FY, JSK, YV, PV and AS conducted the experiments and data analysis; PV and MA designed the mechanical testing protocol. PDP, JL and HVS wrote the manuscript and all authors read and approved the final manuscript.
